# Methylation status of p14ARF and p16INK4a as detected in pancreatic secretions

**DOI:** 10.1038/sj.bjc.6600734

**Published:** 2003-01-28

**Authors:** B Klump, C J Hsieh, O Nehls, S Dette, K Holzmann, R Kießlich, M Jung, U Sinn, M Ortner, R Porschen, M Gregor

**Affiliations:** 1 Department of Internal Medicine I, University Hospital of Tübingen, Otfried-Müller-Street 10, D-72076 Germany; 2 Department of Internal Medicine, St Hildegardis Hospital Mainz, Germany; 3 Department of Internal Medicine, Hospital Bad Cannstatt, Germany; 4 Department of Internal Medicine, Campus Charite Berlin, Germany; 5 Department of Internal Medicine, Central Hospital Bremen East, Germany

**Keywords:** p16, p14, pancreatitis, pancreatic carcinoma, methylation

## Abstract

The clinical management of pancreatic disease is often hampered by a lack of tissue diagnosis. Endoscopic pancreatography offers the opportunity to investigate exfoliated cells. However, the significance of mere cytological investigation is compromised by an insufficient sensitivity. The evaluation of the molecular background of carcinogenesis hopefully is capable of providing more sensitive diagnostic markers. The p16INK4a-/retinoblastoma tumour-suppressive pathway has been shown to be involved in the development of near to all pancreatic neoplasms. p14ARF is another tumour suppressor located in the immediate neighbourhood of p16INK4a. Promoter methylation has been demonstrated to be a major inactivating mechanism of both genes. We sought to further evaluate the role of the gene locus INK4a methylation status in the endoscopic differentiation of chronic inflammatory and neoplastic pancreatic disease. Pancreatic fluid specimens of 61 patients with either pancreatic carcinoma (PCA: 39), chronic pancreatitis (CP: 16) or a normal pancreatogram (NAD: 6) were retrieved. In order to detect methylation of either the p14ARF or the p16INK4a promoter a methylation-specific PCR protocol was applied. While 19 out of 39 patients with PCA showed p16 promoter methylation (49%), none of the 16 patients with CP revealed p16 promoter methylation. p14ARF methylation was found in a lower percentage of PCA specimens and in none of the samples of patients with CP. These results suggest a specific significance of INK4a for the development of malignant pancreatic disease. Our data further indicate a potential role for INK4a methylation as a diagnostic marker in the endoscopic differentiation of benign and malignant pancreatic disease.

The diagnosis of pancreatic cancer in time for cure is still difficult. Clinical symptoms generally only occur in advanced stages of the disease, while no efficient screening tests are available. Furthermore, malignant and benign diseases of the pancreas are difficult to distinguish by even sophisticated imaging procedures or biochemical means requiring surgical diagnosis in a substantial portion of cases. Thus, the prognosis of pancreatic cancer, in spite of improvements, is still fatal, with less than 5% of patients surviving more than 5 years (Niederhuber et al, 1995; Parker et al, 1996).

Pancreatic carcinoma being a ductal disease, sheddering tumour cells with physiological pancreatic secretions into the accessible duodenum has inspired cytology-based diagnostic concepts for decades (Lemon and Byrnes, 1949; Gibbs, 1963). Retrograde pancreatography by a duodenoscope for the first time offered the opportunity to obtain directly ductal juice and to achieve tissue diagnosis in pancreatic disease in a minimal invasive manner (Endo et al, 1974; Hatfield et al, 1976). A multitude of studies have demonstrated that the joint application of endoscopy and cytotechniques has the capability to contribute to an improved clinical management (Tatsuta et al, 1983; Venu et al, 1990; Ryan, 1991; Nakaizumi et al, 1995; Enayati et al, 1996). However, while being characterised by a near 100% specificity, the sensitivity of pancreatic juice cytology proved to be unsatisfying because of an often low cell count or cell disintegration (Ferrari et al, 1994).

Hence, there is a considerable interest in molecular markers that might complement conventional cytotechniques. The demands a molecular candidate marker had to meet were (1) a high prevalence in the tumour under investigation, (2) a high specificity for malignant disease and (3) the methodological possibility to detect reliably those alterations in routinely obtained fluid specimens.

In pancreatic cancer, point mutations of the k-ras oncogene have been shown with a prevalence of up to 90% (Almoguera et al, 1988; Hruban et al, 1993; Urban et al, 1993). Several studies have demonstrated the possibility to detect these mutations in pancreatic juice specimens or duodenal fluid (Tada et al, 1993; Trümper et al, 1994; Berthelemy et al, 1995; Van Laethem et al, 1995). However, further work revealed that k-ras mutations are also associated with benign or precancerous ductal lesions of unclear prognostic significance, thus limiting the clinical significance of detected k-ras mutations (Yanagisawa et al, 1993; Tada et al, 1996; Furuya et al, 1997).

Most human cancer cells are characterised by a disruption of p53- and Rb-tumour suppressive pathways (Sherr, 1996). The uniquely organised ARF/INK4a locus on chromosome 9p harbours two distinct genes, p14ARF and p16INK4a, each one encoding a key member of both pathways, respectively (Chin et al, 1998). Both genes share a common exon, but are under the control of their own specific promoters. The first exon of p14ARF, designated as exon 1*β*, is located approximately 23 kb upstream of the first exon 1*α* of p16INK4a (Quelle et al, 1995; Kamijo et al, 1997). In a variety of human malignancies, p16INK4a alterations apparently are a major cause of Rb pathway impairment. The p16INK4a tumour suppressor gene encodes a cyclin-dependent kinase inhibitor, preferentially binding to CDK4 and CDK6, preventing the coupling of those kinases with d-type cyclins and thus the activating phosphorylation of Rb. Therefore, the functional impairment of p16INK4a is suggested to lead to uncontrolled cell cycle progression and neoplastic transformation (Sherr, 1996; Chin et al, 1998). p14ARF, on the other hand, by accumulating evidence is suggested to act as a tumour suppressor by neutralising MDM2-mediated degradation of p53 (Pomerantz et al, 1998; Stott et al, 1998; Zhang et al, 1998). For pancreatic carcinoma, it has been reported that the Rb tumour-suppressive pathway is abrogated in near to all studied cases and that this disruption is caused exclusively by inactivation of the p16INK4a gene. Herein, aberrant promoter methylation proved to be a major inactivating mechanism (Schutte et al, 1997). While *de novo* promoter methylation of p14ARF has been reported to occur in some gastrointestinal malignancies like stomach and colorectal cancer and precancerous conditions (Barrett et al, 1996; Hsieh et al, 1998; Klump et al, 1998; Esteller et al, 2000; Song et al, 2000; Iida et al, 2000), only a few data exist regarding the situation in pancreatic adenocarcinoma.

The present study sought to further evaluate the significance of aberrant p16INK4a and p14ARF promoter methylation as detected in endoscopically obtained fluid specimens in the differential diagnosis of pancreatic disease. Pancreatic or biliary fluid samples of patients with chronic pancreatitis, pancreatic cancer or missing alterations were analysed with respect to the methylation status of the ARF/INK4a locus in order to define the prevalence and specificity of those epigenetic alterations.

## Patients and Methods

### Patients and specimens

In all, 57 patients in four endoscopic units, who were routinely investigated by endoscopic retrograde cholangiopancreatography (ERCP), were enrolled into this study (37 pancreatic carcinoma (PCA), 14 chronic pancreatitis (CP), six nothing abnormal detected (NAD)). The study was approved by the local ethical committees. The median age of patients with PCA and CP was 70.2 years (39–90 years) and 51.6 years (23–74 years), respectively. The individual diagnosis of pancreatic disease was based on unequivocal histological, biochemical, radiological results and the immediate clinical course. Owing to the often mere palliative treatment of patients with pancreatic adenocarcinoma tissue diagnosis was not available in 18 patients diagnosed to have pancreatic cancer. If a malignant disease other than of pancreatic origin could not definitely be ruled out, samples were excluded from further molecular analysis. Since no standardised protocol in regard to cytological diagnosis was applied and some patients already had a confirmed diagnosis of malignancy when endoscopy was performed, aspirates for cytology were not retrieved in all patients. Thus, malignancy was confirmed in just four patients by cytology. Investigators were asked to try to cannulate the pancreatic duct and aspire pancreatic fluid; in case of technical difficulties it was allowed to either aspire secretions from the Ampulla vateri or—in the case of a supposed infiltration–of the common bile duct. After wire-guided cannulation of the pancreatic duct, between 1 and 3 ml of pancreatic secretion was aspirated. No stimulation by intravenous administration of secretin was performed. No standardised strategy with regard to the exact localisation of the catheter tip was followed. In a few cases, pancreatic fluid was aspired through a prior inserted external drainage of the major pancreatic duct. Immediately after aspiration, obtained specimens were stored without any further processing at −20°C. Additionally, 12 tissue specimens of pancreatic cancer were included. In some patients who were submitted to surgery or had diagnostic biopsy archival tissue specimens, both pancreatic secretions and tissue specimens were analysed.

### DNA extraction

Genomic DNA was extracted from thawed specimens. A volume of 1 ml of secretion was digested for 3 days at 55°C using a proteinase K/sodium dodecyl sulphate (SDS) solution. DNA was extracted using the conventional phenol/chloroform method.

### Methylation-specific PCR amplification

A slight modification of the protocol suggested by Herman et al (1996) has been implemented. In brief, DNA modification by bisulphite converts exclusively unmethylated cytosines to uracil. Subsequent PCR amplification with primers specific for unmethylated vs methylated DNA reveals the methylation status of investigated DNA sections. Initially, 1 *μ*g of DNA was denatured in a volume of 50 *μ*l (final NaOH concentration 0.2 M) for 20 min at 37°C. A total of 30 *μ*l hydroquinone (Sigma, Deisenhofen, Germany) (10 mM) and 520 *μ*l of 3 M sodium bisulphite (Sigma, Deisenhofen, Germany) at pH 5.5 were added and mixed. Samples were incubated at 55°C for 21 h. Modified DNA was purified using a commercially available PCR-Purification Kit according to the manufacturer's recommendations (Qiagen, Hilden, Germany). Finally, a second NaOH treatment was performed (20 min, room temperature, final concentration 0.3 M). Modified and purified DNA was precipitated by ethanol for 12 h and resuspended in 100 *μ*l of water. Primer pairs for PCR amplification are given in Table 1

**Table 1:** Primer pairs

p14 U FW	TTTTTGGTGTTAAAGGGTGGTGTAGT
p14 U RV	CACAAAAACCCTCACTCACAACAA
p14 M FW	GTGTTAAAGGGCGGCGTAGC
p14 M RV	AAAACCCTCACTCGCGACGA
p16 U FW	TTATTAGAGGGTGGGGTGGATTGT
p16 U RV	CAACCCCAAACCACAACCATAA
p16 M FW	TTATTAGAGGGTGGGGCGGATCGC
p16 M RV	GACCCCGAACCGCGACCGTAA

U=specific for unmethylated DNA , M=specific for methylated DNA.

and were purchased (MWG-Biotech, Ebersberg, Germany). A volume of 100 *μ*l PCR mixtures contained 10 *μ*l buffer (10 mM Tris-HCl, 50 mM KCl, 0.1% Triton X-100), 1 *μ*l of MgCl_2_, 1.5 *μ*l of dNTPs (1.25 mM), primers, 2 U of *Taq* polymerase (PAN-Systems, Aidenbach, Germany), and 0.1 *μ*g of DNA. Amplification was performed in a thermal cycler (Biometra, Göttingen, Germany) for 35 cycles (95°C/5 min, annealing temperature/90 s, 72°C/60 s) and concluded by a final 8 min extension at 72°C. A control without the addition of DNA was performed for each PCR set. A volume of 20 *μ*l of PCR product was loaded onto nondenaturing polyacrylamide gels (8%) and visualised by silver staining. A methylation was confirmed if at least two experiments had demonstrated an unequivocal amplification product of methylation-specific PCR. Around 10 consecutive specimens were analysed as common slots without clinical information on singular specimens.

### Statistical analysis

The association of p16INK4a and p14ARF promoter methylation in malignant vs nonmalignant pancreatic disease and patients with a normal pancreatogram was tested by *χ*^2^ test and Yates' correction.

## Results

### p16-promoter methylation

**Figure 1:**
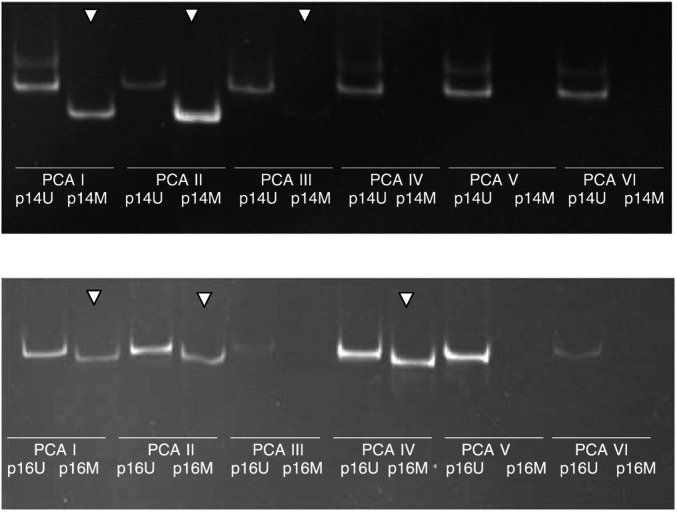
Gels with products of PCR specific for either an unmethylated (U) or a methylated (M) promoter region

In total, 43.2% of specimens obtained from patients with pancreatic carcinoma revealed p16 promoter methylation (16 out of 37). However, none out of 16 specimens obtained from patients with chronic pancreatitis (*n*=14) or missing alterations (*n*=6) showed p16 promoter methylation. Thus, a sensitivity of 43.2% and a specificity of 100% in regard to the detection of malignancy has been found for p16 promoter methylation (Figure 1

, Table 2

**Table 2:** Methylation data within pancreatic secretions and/or tissue specimens

		**Pancreatic secretion**		**Tissue**	
**No.**	**Diagnosis**	**p14M**	**p16M**	**p14M**	**p16M**
1	PCA	n	n	n	n
2	PCA	n	n	n	n
3	PCA	n	y	n	n
4	PCA	n	n	y	y
5	PCA	n	n	n	y
6	PCA	nd	n	n	n
7	PCA	nd	nd	n	n
8	PCA	nd	nd	n	n
9	PCA	nd	nd	N	n
10	PCA	nd	nd	y	n
11	PCA	nd	nd	y	y
12	PCA	nd	nd	n	n
13	PCA	n	n	nd	nd
14	PCA	nd	n	nd	nd
15	PCA	y	y	nd	nd
16	PCA	n	n	nd	nd
17	PCA	n	y	nd	nd
18	PCA	n	y	nd	nd
19	PCA	n	y	nd	nd
20	PCA	n	n	nd	nd
21	PCA	n	y	nd	nd
22	PCA	y	y	nd	nd
23	PCA	n	n	nd	nd
24	PCA	n	y	nd	nd
25	PCA	n	n	nd	nd
26	PCA	n	n	nd	nd
27	PCA	nd	y	nd	nd
28	PCA	n	y	nd	nd
29	PCA	y	y	nd	nd
30	PCA	y	n	nd	nd
31	PCA	y	n	nd	nd
32	PCA	n	y	nd	nd
33	PCA	n	n	nd	nd
34	PCA	n	n	nd	nd
35	PCA	y	y	nd	nd
36	PCA	n	n	nd	nd
37	PCA	n	y	nd	nd
38	PCA	n	n	nd	nd
39	PCA	n	n	nd	nd
40	PCA	y	y	nd	nd
41	PCA	n	n	nd	nd
42	PCA	n	n	nd	nd
43	PCA	n	y	nd	nd
44	CP	n	n	nd	nd
45	CP	n	n	nd	nd
46	CP	n	n	nd	nd
47	CP	n	n	nd	nd
48	CP	n	n	nd	nd
49	CP	n	n	nd	nd
50	CP	n	n	nd	nd
51	CP	n	n	nd	nd
52	CP	n	n	nd	nd
53	CP	n	n	nd	nd
54	CP	n	n	nd	nd
55	CP	n	n	nd	nd
56	CP	n	n	nd	nd
57	CP	n	n	nd	nd
58	NAD	n	n	nd	nd
59	NAD	n	n	nd	nd
60	NAD	n	n	nd	nd
61	NAD	n	n	nd	nd
62	NAD	n	n	nd	nd
63	NAD	n	n	nd	nd

and Table 3

**Table 3:** Results of methylation analysis (p14M, p16M) in secretions for pats without clinical signs of pancreatic disease (NAD), chronic pancreatitis (CP), or pancreatic carcinoma (PCA)

	**NAD**	**CP**		**PCA**	
*p14M*	0/6 (0%)	0/14 (0%)		7/34 (20.6%)	
		*P*>0.1 (*χ*^2^=3.16)/n.s.			
		*Pancreatic duct*	*Other tip localisation*	*Pancreatic duct*	*Other tip localisation*
		0/12	0/2	2/13	5/21
*p16M*	0/6 (0%)	0/14 (0%)		16/37 (43.2%)	
		*P*<0.01 (*χ*^2^=11.91)			
		*Pancreatic duct*	*Other tip localisation*	*Pancreatic duct*	*Other tip localisation*
		0/12	0/2	6/14	10/23

).

### p14-promoter methylation

Methylation of the p14 promoter was detected in 20.6% of specimens of patients with pancreatic carcinoma (seven out of 34). Promoter methylation of p14ARF could neither be detected in specimens obtained from patients with chronic pancreatitis nor in those samples from patients with missing alterations. Thus, a sensitivity of 20.6% and a specificity of 100% in regard to the detection of malignancy has been shown for p14 promoter methylation (Figure 1, Table 2 and Table 3).

### Relation of p14 and p16 promoter methylation

A simultaneous promoter methylation of p14ARF and p16INK4a was seen in 14.7% of the specimens from patients with pancreatic carcinoma. A simultaneous methylation of the adjacent p14ARF promoter was seen in 22% of those specimens with positive p16INK4a methylation (Table 2). An exclusive methylation of either the p16INK4a or the p14ARF promoter region was observed in 72 and 43% of methylation-positive carcinoma specimens, respectively.

## Discussion

Molecular approaches keep the promise of complementing cytotechniques in the noninvasive diagnosis of pancreaticobiliary disease. The potential sensitivity of any molecular alteration, however, is defined by its prevalence in pancreatic cancer, its specificity for malignant alterations and the technical options to detect it reliably.

The methodological success of PCR-based approaches towards the molecular analysis of pancreatic fluids is limited by polymerase inhibitors. Like other groups, we observed the frequent failure of PCR analysis in pilot studies (Saurin et al, 2000). In our experience, it is essential to find out the least possible amount of DNA allowing PCR amplification. However, our preliminary studies revealed that methylation analysis, even though requiring additional processing steps and being a gradual process, is even more reproducible in endoscopically obtained secretions than mutational screening assays as PCR-SSCP or PCR-RFLP. The present study is the first to demonstrate the diagnostic potential of INK4a methylation in pancreatic secretions obtained during routine endoscopic retrograde pancreatography. Methylation status of both the p14ARF and the p16INK4a promoter was successfully assessed in all samples enclosed. Surprisingly, unlike the results of a previous preliminary analysis, the localisation of the catheter tip did not influence the prevalence of detected methylation. We speculate that exfoliated cells were not only present in the pancreatic duct or Ampulla vateri, but also in the common bile duct due to an infiltration of the biliary system with a ‘double duct sign’ detectable in most cases. However, it is to be assumed that due to a very small number of patients in our study a difference in technical approaches towards aspiration of secretions was missed.

Interestingly, the methylation rates detected in tissue proved to be somewhat lower than those detected in endoscopically retrieved pancreatic secretions. While it seems intriguing to speculate on whether fluid samples retrieved from the pancreatic duct might be even more representative than tissue slices from tumour blocks, it has to be taken into consideration that (1) the numbers of patients in whom tissue could be analysed were small and (2) no standardised protocol in regard to the retrieval and storage of tissue specimens was followed; detected methylation rates could be lower due to degradation of DNA and/or contamination with benign ductal or stromal cells in archival paraffin blocks.

*De novo* p16INK4a promoter methylation was detected in 43.2% of specimens from patients with pancreatic carcinoma. These data parallel those recently reported by Schutte et al (1997), who detected p16INK4a promoter methylation in six out of 17 primary pancreatic carcinoma specimens or pancreatic carcinoma cell lines (35%). Similarly, a prevalence of p16INK4a methylation of 38% has recently been reported by Esteller et al (2001). In this context, it needs to be emphasised that exclusively exocrine pancreatic adenocarcinomas of ductal origin have been included. A recent study published by Moore et al (2001) has addressed p16 alterations in primary neoplasms of the pancreas, and these data have shown that in exocrine and endocrine tumourigenesis of the pancreas different molecular pathways may be involved.

The situation for p14ARF is more controversial. As far as we know, up to this end the only data regarding the role of p14ARF in pancreatic carcinoma have been published also by Esteller et al (2000): in none out of 20 randomly selected pancreatic carcinomas p14ARF promoter methylation was detected. This is in apparent contrast to our findings, showing a prevalence of at least 20.6% in 36 pancreatic fluid specimens. While it is well recognised that promoter methylation is a gradual process, our results could reflect a weak methylation of unknown functional significance. In addition, a ‘background level of methylation’ had also been reported by Schutte et al (1997) in normal duodenal tissue. Moreover, there is an ongoing controversy as to whether age-related methylation possibly may also involve promoter regions of tumour-suppressive genes. It is to be acknowledged that patients with pancreatic carcinoma tend to be older than patients with chronic pancreatitis. Thus, a contribution of age-related methylation may not be ruled out when higher rates of promoter methylation are detected in neoplasms. And indeed in this study, for p16 promoter methylation a lower frequency in patients 63 years and younger was detected, while patients of 80 years and older revealed a methylated promoter more frequently than the group as a whole. However, for p14 methylation in older patients an even lower frequency of methylation was detected (data not shown).

Taken together, at the moment it may be postulated that p14ARF promoter methylation is at least rarer than p16INK4a methylation in pancreatic carcinoma and that further evaluation of p14ARF's exact role in the development and course of pancreatic carcinoma requires further studies in greater patient cohorts, bringing together methylation data with functional findings.

In none of the specimens obtained from patients with chronic pancreatitis, either p16INK4a or p14ARF promoter methylation was detected in this study. This further supports the results of our own previous work and the data reported by others indicating that the tumour-suppressive function of p16INK4a is exclusively abrogated in (pre-)neoplastic but not in merely inflammatory or hyperproliferative conditions (Barrett et al, 1996; Hsieh et al, 1998; Klump et al, 1998). However, it is well recognised from epidemiological and histopathological studies that chronic pancreatitis may predispose for the development of pancreatic cancer. Thus, the prevalence of INK4a methylation in preneoplastic ductal lesions requires further evaluation. For pancreatic disease our findings are in fortunate contrast to the situation found for k-ras mutations and suggest the further evaluation of INK4a methylation in the management of unclear pancreatic disease.

Taken together, our findings for the first time demonstrate the technical feasibility to detect INK4a's methylation in pancreatic secretions. Moreover, the high prevalence of p16INK4a methylation found in pancreatic carcinoma tissues is confirmed by these data. INK4a methylation revealed a 100% specificity for malignant pancreatic disease in this study. p14ARF methylation apparently is of subordinate significance in pancreatic carcinoma while its exact role needs to be further evaluated.

We would conclude, therefore, that INK4a methylation is a promising candidate marker for the endoscopic differential diagnosis of malignant and benign pancreatic disease.
